# Genetic screening in a Brazilian cohort with inborn errors of immunity

**DOI:** 10.1186/s12863-023-01148-z

**Published:** 2023-08-17

**Authors:** Cristina Santos Ferreira, Ronaldo da Silva Francisco Junior, Alexandra Lehmkuhl Gerber, Ana Paula de Campos Guimarães, Flavia Amendola Anisio de Carvalho, Bárbara Carvalho Santos dos Reis, Fernanda Pinto-Mariz, Monica Soares de Souza, Zilton Farias Meira de Vasconcelos, Ekaterini Simões Goudouris, Ana Tereza Ribeiro Vasconcelos

**Affiliations:** 1 Bioinformatics Laboratory-LABINFO, National Laboratory of Scientific Computation LNCC/MCTIC, Av. Getúlio Vargas, 333, Quitandinha CEP: 25651-075, Petrópolis, Rio de Janeiro Brazil; 2 Allergy and Immunology Service of Institute of Women, Children and Adolescents’ Health Fernandes Figueira (IFF/FIOCRUZ), Rio de Janeiro, RJ Brazil; 3 Allergy and Immunology Service of the Martagão Gesteira Institute for Childcare and Pediatrics (IPPMG) - Federal University of Rio de Janeiro (UFRJ), Rio de Janeiro, RJ Brazil; 4 Allergy and Immunology Sector of the Pediatric Service of the Federal Hospital of Rio de Janeiro State (HFSE) – Ministry of Health, Rio de Janeiro, RJ Brazil; 5 Laboratory of High Complexity of the Institute of Women, Children and Adolescents’ Health Fernandes Figueira (IFF/FIOCRUZ), Rio de Janeiro, RJ Brazil

**Keywords:** Inborn errors of immunity, Whole exome sequencing, Genetic screening, Single nucleotide variants

## Abstract

**Background:**

Inherited genetic defects in immune system-related genes can result in Inborn Errors of Immunity (IEI), also known as Primary Immunodeficiencies (PID). Diagnosis of IEI disorders is challenging due to overlapping clinical manifestations. Accurate identification of disease-causing germline variants is crucial for appropriate treatment, prognosis, and genetic counseling. However, genetic sequencing is challenging in low-income countries like Brazil. This study aimed to perform genetic screening on patients treated within Brazil's public Unified Health System to identify candidate genetic variants associated with the patient’s phenotype.

**Methods:**

Thirteen singleton unrelated patients from three hospitals in Rio de Janeiro were enrolled in this study. Genomic DNA was extracted from the peripheral blood lymphocytes of each patient, and whole exome sequencing (WES) analyses were conducted using Illumina NextSeq. Germline genetic variants in IEI-related genes were prioritized using a computational framework considering their molecular consequence in coding regions; minor allele frequency ≤ 0.01; pathogenicity classification based on American College of Medical Genetics and Genomics and the Association for Molecular Pathology (ACMG/AMP) guidelines gathered from the VarSome clinical database; and IEI-related phenotype using the Franklin tool. The genes classification into IEI categories follows internationally recognized guidelines informed by the International Union of Immunological Societies Expert Committee. Additional methods for confirmation of the variant included Sanger sequencing, phasing analysis, and splice site prediction.

**Results:**

A total of 16 disease-causing variants in nine genes, encompassing six different IEI categories, were identified. X-Linked Agammaglobulinemia, caused by *BTK* variations, emerged as the most prevalent IEI disorder in the cohort. However, pathogenic and likely pathogenic variants were also reported in other known IEI-related genes, namely *CD40LG*, *CARD11*, *WAS*, *CYBB*, *C6*, and *LRBA*. Interestingly, two patients with suspected IEI exhibited pathogenic variants in non-IEI-related genes, *ABCA12* and *SLC25A13*, potentially explaining their phenotypes.

**Conclusions:**

Genetic screening through WES enabled the detection of potentially harmful variants associated with IEI disorders. These findings contribute to a better understanding of patients' clinical manifestations by elucidating the genetic basis underlying their phenotypes.

**Supplementary Information:**

The online version contains supplementary material available at 10.1186/s12863-023-01148-z.

## Background

Inborn errors of Immunity (IEI) are a broad group of inherited immune system disorders leading to enhanced susceptibility to infections. Other conditions include autoimmunity, autoinflammatory diseases, atopic manifestations, and hematopoietic or solid tissue malignancies [1]. These monogenic illnesses are often caused by deleterious germline variants in immunity-related genes [2, 3], with an estimated frequency of one case for every 10,000 patients in Latin America [4]. Nevertheless, as novel IEI disorders continue to be discovered and clinical phenotypes are better defined, the cumulative prevalence worldwide is expected to be at least 1 in 1000 to 1 in 5000 [1]. Currently, 485 IEI were already identified, which are divided into ten different categories [3, 5]. Approximately 452 loci across the human genome were involved in the pathogenesis of IEI [5]. Several other inheritance models and molecular mechanisms were observed among the known IEI phenotypes. IEI categories were assigned based on each disease's clinical manifestations, immunological alterations, and laboratory findings. Considering the heterogeneous clinical features, the diagnosis of IEI is challenging, leading to misdiagnosis in some cases [6]. An assertive genetic diagnosis is usually achieved in ~ 40% of cases indicating the complexity and heterogeneity of IEI [7].

Advances in molecular genetics and cellular immunology allowed a much better resolution of several IEI categories. Consequently, speeding up the prognostic predictions and contributing to the patient’s management [6, 8, 9]. Studies using massively parallel sequencing technologies, including whole exome sequencing (WES) or whole genome sequencing (WGS) were able to increase the diagnostic yields from 15 to 79%, allowing new therapies, genetic counseling, and identification of new disease-related genes [10–13]. Given that the majority of disease-causing variants in monogenic disorders are found in protein-coding regions [14], despite limitations, WES is an affordable approach for diagnosing rare genetic disorders, displaying crucial advances compared to basic Sanger sequencing, panel-target sequencing, or the broader screening provided by WGS [7, 15, 16]. The WES limitations include the inability to detect variants in regions with less coverage or located in the promoter, regulator, or intronic regions; additionally, detecting complex structural variations through next-generation DNA sequencing can be quite challenging [15, 17, 18].

Although WES has already been implemented in the clinical routine of many countries, in Brazil, this approach is still limited to private companies and research studies. The limited availability of resources to provide a personalized screening of IEI variants has left many patients and family members without an assertive genetic diagnosis. Considering the aspects of monogenic disorders, their kindreds are also affected by lacking genetic counseling. Such differences could be even higher by looking at the underreporting issues faced by the public Unified Health System (“Sistema Único de Saúde” or SUS) across the distinct Brazilian regions. In this study, a genetic variant prioritization was performed on 13 patients treated at SUS hospital facilities. The objective was to provide a personalized screening of candidate germline variants potentially associated with their clinical manifestations and suspicion of IEI.

## Results

### Description of cohort

The 13 singleton patients included in this study presented clinical manifestations established in the inclusion criteria that led to the suspicion of an IEI at different lifetimes, supported by multiple clinical or laboratory tests. The cohort comprised 12 (92.3%) males and one (7.7%) female from unrelated Brazilian families in Rio de Janeiro (Table S1, and Figure S1). On average, patients presented the onset of the symptoms in early childhood, from the first year of life, varying from birth to five years old (y.o.). The median age was 10 y.o. (interquartile range 6–12 y.o., range 1–17 y.o.) (Table S1, and Figure S1). The patients presented immunological impairment, followed by recurrent infection episodes, remarkable pulmonary impairment, and/or altered levels of specific immunoglobulin production. In two patients, the microorganism culture showed secondary infection caused by *Pseudomonas sp.*, and *Klebsiella sp..* The most common infections were pneumonia (37.5%), sinusitis (20.8%), otitis (16.7%), and arthritis (12.5%) (Figure S1). Though less frequently, urinary tract infections (4.2%), meningitis 4.2%), and encephalitis (4.2%) were also noticed. For one patient, microabscesses on the liver without a known infectious cause were reported and characterized as autoimmunity and another single patient had a phenotype consistent with complex ichthyosis condition and severe chickenpox case.

### Genetic findings and candidate damage variants

**Fig. 1:**
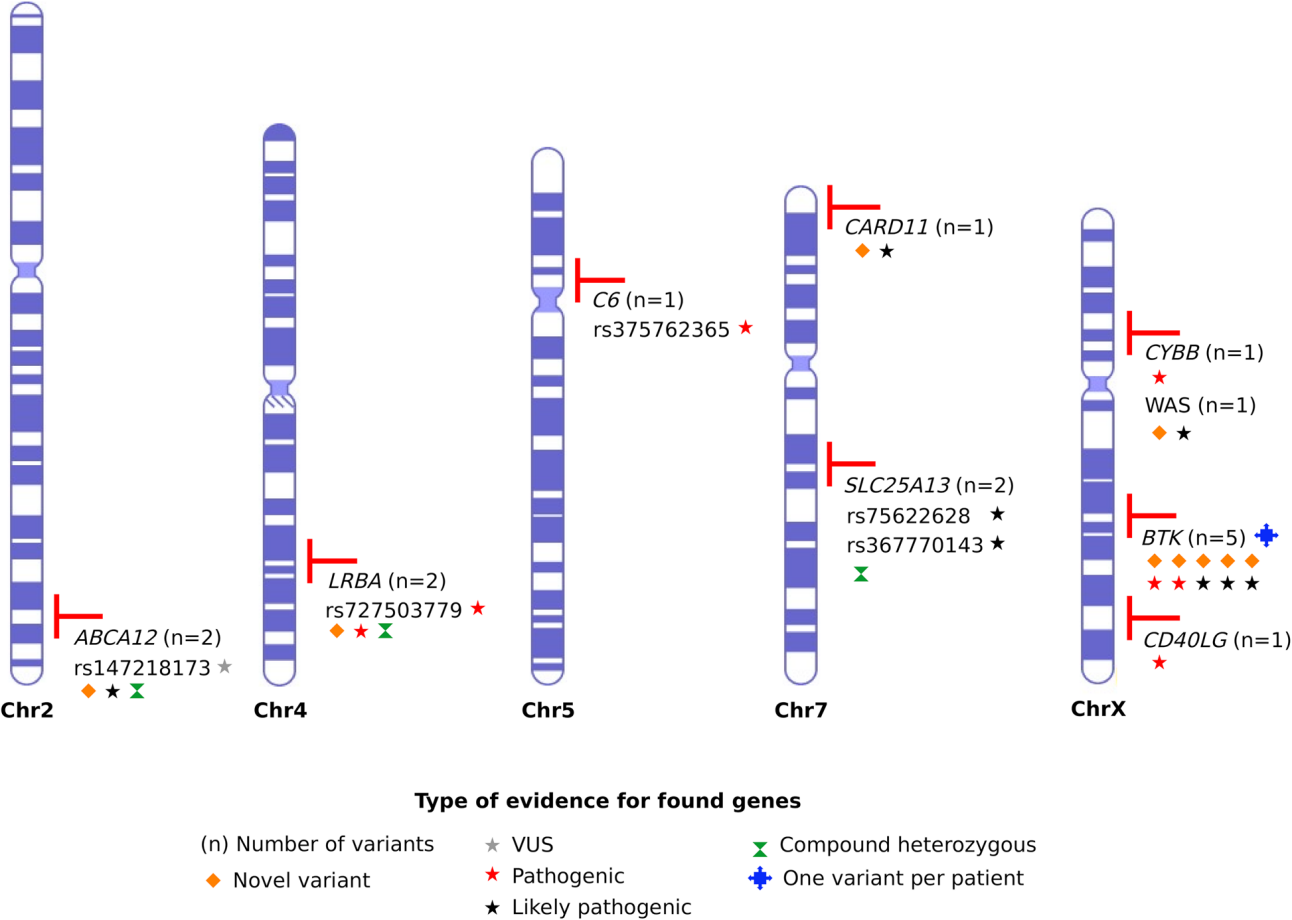
Genome-wide distribution of genes harboring the harmful variants prioritized. Distribution of the main genes and variants across chromosomes. Gray, red or black stars represent VUS, pathogenic or likely pathogenic variants, respectively. The number of variants per gene is summarized beside each gene symbol. The compound heterozygous genotype is represented by a green symbol and the multiple patients per gene are shown as a blue symbol

The genetic screening in the studied cohort revealed the presence of pathogenic, likely pathogenic, and/or variants of uncertain significance (VUS) associated with genetic disorders in 13 cases (Table 1, Table S2). Only VUS candidates to have a genotype–phenotype correlation that could impact the clinical manifestations of the patients were reported. However, further studies are needed to confirm these VUS as responsible for the clinical phenotype. For all patients, additional non-IEI-related variants were found (Table S2). The 16 single nucleotide variants (SNVs) found in the 13 patients overlapped nine genes, of which seven were well-characterized to cause IEI, including *BTK, CD40LG, CARD11, WAS, CYBB, C6, and LRBA* (Table 1 and Table S2). The nine genes were mainly distributed among the chromosomes 2 (11.1%), 4 (11.1%), 5 (11.1%), 7 (22.2%), and X (44.4%), as shown in Figure S1 and Fig. 1. Most phenotypes associated with the genes displayed an X-linked recessive (XLR; 53.8%) inheritance pattern, followed by autosomal recessive (AR; 30.8%) and autosomal dominant (AD; 7.6%) models (Figure S1). Moreover, missense (31.25%), frameshift (25%), nonsense (12.5%), splicing (12.5%), and in-frame insertions or deletions (6.25%) were among the most frequent molecular consequences observed in the pathogenic/likely pathogenic (P/LP) variants (Figure S1). Among the IEI categories previously established by the International Union of Immunological Societies (IUIS) Expert Committee, the candidate disease-causing variants in the 13 patients were mainly associated with predominantly antibody deficiencies (38.5%), followed by immunodeficiencies affecting cellular and humoral immunity (15.4%), combined immunodeficiencies with associated or syndromic features (7.7%), congenital defects of phagocyte number or function (7.7%), complement deficiencies (7.7%), and regulatory T cell defect (7.7%). It was also observed that 15.4% of the identified variants did not match any of the genes listed in the IUIS categories. However, according to the analysis, these variants were deemed relevant to the patient’s phenotype (Figure S1).

**Table 1:** Potentially IEI-causative variants identified in patients WES data

Patient	OMIM Disease	OMIM number	Inheritance	Gene	Variant	Zigosity	MAF	ACMG Classification
Patient 1	Agammaglobulinemia, X-linked 1	300755	XLR	*BTK*	NM_000061.3:c.1111_1112dup (p.Arg372ProfsTer32)	Hem		LP
Patient 2	Agammaglobulinemia, X-linked 1	300755	XLR	*BTK*	NM_000061.3:c.167T > A (p.Ile56Lys)	Hem		LP
Patient 3	Agammaglobulinemia, X-linked 1	300755	XLR	*BTK*	NM_000061.3:c.993dup (p.Arg332ThrfsTer17)	Hem		LP
Patient 4	Agammaglobulinemia, X-linked 1	300755	XLR	*BTK*	NM_000061.3:c.336C > A (p.Tyr112Ter)	Hem		P
Patient 5	Agammaglobulinemia, X-linked 1	300755	XLR	*BTK*	NM_000061.3:c.1735G > T (p.Asp579Tyr)	Hem		P
Patient 6	Immunodeficiency, X-linked, with hyper-IgM	308230	XLR	*CD40LG*	NM_000074.3:c.436_438del (p.Tyr146del)	Hem		P
Patient 7	B-cell expansion with NFKB and T-cell anergy	616452	AD	*CARD11*	NM_032415.7:c.752T > C (p.Leu251Pro)	Het		LP
Patient 8	Wiskott-Aldrich Syndrome	301000	XLR	*WAS*	NM_000377.3:c.889C > T (p.Gln297Ter)	Hem		LP
Patient 9	Chronic granulomatous disease, X-linked	306400	XLR	*CYBB*	NM_000397.4:c.483G > A (p.Lys161 =)	Hem		P
Patient 10	C6 deficiency	612446	AR	*C6*	NM_001115131.3:c.1138del (p.Gln380SerfsTer7)	Hom	0.000514112	P
Patient 11	Immunodeficiency, common variable, 8, with autoimmunity	614700	AR	*LRBA*	NM_001367550.1:c.6624_6625del(p.Glu2208AspfsTer3)	Het		P
					NM_001367550.1:c.7452+1G > T			﻿P
Patient 12	Ichthyosis, congenital, autosomal recessive 4B (harlequin)	242500	AR	*ABCA12*	NM_173076.3:c.318-2A > G	Het		LP
					NM_173076.3:c.2033A > G (p.Asn678Ser)		0.00137	VUS
Patient 13	Citrullinemia, type II, neonatal-onset	605814	AR	*SLC25A13*	NM_014251.3:c.1618C > T(p.Pro540Ser)	Het	0.000103421	LP
					NM_014251.3:c.1754G > A(p.Arg585His)		0.0000119439	LP

*AR* Autosomal recessive, *AD* Autosomal dominant, *XLR* X-linked recessive, *Hem* Hemizygous, *Hom* Homozygous, *Het* Heterozygous, *MAF* minor allele frequency, *LP* Likely Pathogenic, *P* Pathogenic, *VUS* Variant of Uncertain Significance

### Genotype–phenotype correlations

#### Predominantly antibody deficiencies

Five unrelated male patients from nonconsanguineous couples were identified with symptoms consistent with X-linked Agammaglobulinemia (XLA; OMIM #300755) harboring SNVs in the *BTK* gene (Table 1 and Table S2). *BTK* gene encodes a non-receptor tyrosine kinase protein composed of five different domains, including the N-terminal pleckstrin homology (PH), the TEC homology (TH), SRC homology 3 (SH3), SH2, and the Tyrosine Kinase (TK) domain at the C-terminal region [19, 20]. The five SNVs found across the different patients overlapped the PH (*n* = 2), SH2 (*n* = 2), and TK (*n* = 1) domains. The subjects presented reduced levels of all immunoglobulins, CD19 and CD20. They also showed recurrent infections in early childhood with pneumonia, sinusitis, otitis, arthritis, and, less frequently, subacute encephalitis phenotype (Table 2).

**Table 2:** Clinical IEI-manifestations of agammaglobulinemia patients harboring candidate disease-causing variants in *BTK* gene

Patient number	Reccurent infections	Hematological alteration					Variant in *BTK* gene	BTK domain
		**Blood cell count (%)**	**Immunoglobulin level**					
			**IgA (mg/dL)**	**IgG (mg/dL)**	**IgM (mg/dL)**	**IgE (UI/L)**		
Patient 1	Sinusite, Pneumonia, and *Pseudomonas sp*. infection	CD19, CD20 undetectable	undetectable	579	undetectable	undetectable	NM_000061.3:c.1111_1112dup (p.Arg372ProfsTer32)	SH2
				RV: 553–971				
Patient 2	Pneumonia, Otite, Sinusite	CD19 = 0.7	8	65	32	undetectable	NM_000061.3:c.167T > A (p.Ile56Lys)	PH
		RV: 6–23	RV: 4–7	RV: 338–406	RV: 29–32			
Patient 3	Artrite, Pneumonias, Sinsuites	CD19 less than 1	17.3	44.5	8.9	0.5	NM_000061.3:c.993dup (p.Arg332ThrfsTer17)	SH2
		RV: 6–23	9	RV: 739–793	RV: 81–92	RV: 0 -0.1		
Patient 4	Otite, Sinusite, Pneumonia, Artrite	CD19 and CD20 = 0.2	undetectable	< 30	57	undetectable	NM_000061.3:c.336C > A (p.Tyr112Ter)	PH
		RV: 13–27; RV: 7.1–23.8		RV: 616–799	RV: 64–87			
Patient 5	Chronic arthritis, pneumonia, and bronchiectasis	CD19 = 0.4; CD20 = 0.2	undetectable	12	undetectable	undetectable	NM_000061.3:c.1735G > T (p.Asp579Tyr)	TK
		RV: 13–27; RV: 7.7–24.3		RV: 338–406				

*RV* Reference Value, *SH2* SRC homology 2, *PH* Bruton's tyrosine kinase pleckstrin homology, *TK* Catalytic domain of the Protein Tyrosine Kinases

Patient 1 is a 15 y.o. male, with a family history of IEI in a male cousin, harbored a likely pathogenic frameshift variant NM_000061.3:c.1111_1112dup (p.Arg372ProfsTer32). In patient 2, a 16 y.o. male with a family history of maternal uncles who died from infections, a likely pathogenic missense variant NM_000061.3:c.167T > A (p.Ile56Lys) was reported. Patient 3 is a 17 y.o. male presented with oral polio vaccine reaction and subacute encephalitis, which caused his death at 18 years old. The likely pathogenic frameshift NM_000061.3:c.993dup (p.Arg332ThrfsTer17) was found. Patient 4 is a 12 y.o. male harbored a pathogenic nonsense alteration NM_000061.3:c.336C > A (p.Tyr112Ter). Finally, patient 5 is a seven y.o. male diagnosed with meningocele at birth submitted to surgery. His familial history shows early death of maternal uncles from unknown causes. A pathogenic missense SNV NM_000061.3:c.1735G > T (p.Asp579Tyr) was found (Table 1 and Table S2).

#### Immunodeficiencies affecting cellular and humoral immunity

Two candidate damage variants were identified in *CD40LG* and *CARD11* genes previously associated with Combined Immunodeficiency (CID). Both subjects presented reduced levels of immunoglobulins and recurrent infections in early childhood with pneumonia, sinusitis, otitis (acute otitis media), and pyoderma.

Patient 6 is a 10 y.o. male with a family history showing a brother's death from unknown causes. He also presented agammaglobulinemia phenotype, and hyper-IgM syndrome with Glucose-6-phosphate dehydrogenase deficiency (G6PD). A pathogenic hemizygous in frame deletion NM_000074.3:c.436_438del (p.Tyr146del) was found in the *CD40LG* gene related to Immunodeficiency, X-linked, with hyper-IgM (OMIM ﻿#308230). Patient 7 is a 10 y.o. male with the onset of the symptoms after one and a half years of life with lymphoproliferation and B lymphocytosis. A likely pathogenic missense variant NM_032415.7:c.752T > C (p.Leu251Pro) was identified in the *CARD11* gene. *CARD11* is associated with B cell expansion with NF-κB and T cell anergy (OMIM #616452) (Table 1 and Table S2).

#### Combined immunodeficiencies with associated or syndromic features

A single patient harbored a harmful SNV in the *WAS* gene associated with immunodeficiency with congenital thrombocytopenia. Patient 8 is a one-year-old male who first showed symptoms of thrombocytopenia with small platelets besides chronic diarrhea with digestive bleeding at four months of age. He was first misdiagnosed as food allergy with an additional diagnosis of Inflammatory Bowel Disease. He carried a likely pathogenic hemizygous nonsense variant NM_000377.3:c.889C > T (p.Gln297Ter) in the *WAS* gene related to Wiskott-Aldrich Syndrome (OMIM #301000) (Table 1 and Table S2).

#### Congenital defects of phagocyte number or function

Variants in the *CYBB* gene have been associated with respiratory burst defects among congenital defects of phagocyte number. A pathogenic SNV was identified in *CYBB* in a single patient in the cohort. Patient 9 is a four y.o. male with a family history showing a brother's death from unknown causes. His symptoms started during the second month of life with recurrent pneumonia, anemia, and urinary tract infection caused by *Klebsiella spp*. A pathogenic hemizygous synonymous variant NM_000397.4:c.483G > A (p.Lys161 =) was found in the *CYBB* gene related to Chronic granulomatous disease, X-linked (OMIM #306400) with XLR inheritance, that characterizes his Chronic granulomatous disease phenotype (Table 1 and Table S2). A splice region prediction analysis was performed for the variant region. It indicated a broken splice site alteration, particularly in a prospective 5′ donor splice site, evoking suspicions that it may affect gene function and protein synthesis (Table S3).

#### Complement deficiencies

A rare pathogenic SNV was found in the *C6* gene related to complement deficiencies. Patient 10 is a nine y.o. male of couples claiming to be nonconsanguineous with clinical manifestations compatible with complement disease. His symptoms onset was observed at five y.o. with low rates of CH50 and recurrent meningitis. He had a sister who was also diagnosed with meningitis by unknown causes. The homozygous pathogenic frameshift variant NM_001115131.3:c.1138del (p.Gln380SerfsTer7) was found in the *C6* gene associated with C6 deficiency (OMIM #612446) (Table 1 and Table S2). Trio sequencing including samples from both parents and patient 10 was performed to validate the recessive inheritance pattern of frameshift variant p.Gln380SerfsTer7 in the patient. Both parents were heterozygous for the variant and the patient’s homozygosity was confirmed. Supplementary Figure S2 shows the electropherogram of Sanger sequencing and the visualization of Next Generation Sequencing (NGS) reads in the locus.

#### Regulatory T Cell defect

Two rare pathogenic variants in the *LRBA* gene associated with T cell dysregulation were identified in patient 11. Recurrent infections, inflammatory bowel disease, and autoimmunity characterize this group of disorders. The patient is 11 y.o. female with symptoms onset at the third year of life with autoimmune manifestations leading to systemic myasthenia gravis, celiac disease, insulin-dependent diabetes mellitus, severe pulmonary infection, pancytopenia/bicytopenia, hemorrhagic stroke and hypothyroidism. WES analysis revealed two SNVs in heterozygosis in the *LRBA* gene related to Immunodeficiency, common variable, 8, with autoimmunity, AR (OMIM #614700). The NM_001367550.1:c.6624_6625del (p.Glu2208AspfsTer3) is a frameshift and the second variant NM_001367550.1:c.7452+1G > T is in a splicing site. Both SNVs may suggest a compound heterozygous genotype (Table 1 and Table S2). The reconstruction of variant phases using NGS reads proved unfeasible due to their genetic distance.

#### Phenotypes associated with variants in non-IEI-related genes

For two patients previously included in this study with suspicion of IEI, it was infeasible to identify candidate SNVs in genes included in the 2022 classification of the International Union of Immunological Societies Expert Committee. Nevertheless, it was possible to identify SNVs associated with the subject’s phenotype, and the zygosity profile of the variants matched the inheritance pattern of the disease.

Patient 12 is six y.o. male with symptoms being observed at birth characterized as a harlequin fetus. He presented recurrent episodes of pneumonia during the first year of his life, besides severe episodes of chickenpox, and complex ichthyosis manifestation. Two SNVs in the *ABCA12* gene related to Ichthyosis, congenital, autosomal recessive 4B (harlequin) (OMIM #242500) were identified. The NM_173076.3:c.318-2A > G is a splicing site variant classified as likely pathogenic by the American College of Medical Genetics and Genomics and the Association for Molecular Pathology (ACMG/AMP) guidelines. The second variant NM_173076.3:c.2033A > G (p.Asn678Ser), had a missense effect, being classified as VUS (Table 1 and Table S2). Both SNVs may suggest a compound heterozygous genotype. The phasing association between heterozygous variants was infeasible due to their genetic distance. Patient 13 is one y.o. male with symptoms onset being observed after six months old mirroring the clinical signs of autoimmunity with persistent fever, and recurrent microabscesses in the liver, without an infectious agent. Additionally, he presented bicytopenia, polyclonal hypergammaglobulinemia, and Evans syndrome. Two likely pathogenic heterozygous missense SNVs NM_014251.3:c.1618C > T (p.Pro540Ser) and NM_014251.3:c.1754G > A (p.Arg585His) were in the *SLC25A13* gene related to autosomal recessive Citrullinemia, type II, neonatal-onset (OMIM #605814) (Table 1 and Table S2). The phasing analysis revealed that these variants were in different chromosomes, suggesting a compound heterozygous genotype in trans configuration (Table S4).

## Discussion

This study describes the genetic screening conducted on 13 patients suspected to have an IEI. The recurrent infection episodes in early childhood and altered immunological findings raised concerns about the possibility of IEI-related disorders in the cohort [21, 22]. Many infection episodes were associated with a reduced number or function of one or more immunological cell types, as previously described in IEI patients [23, 24]. However, this broad group of disorders often shares common clinical manifestations which challenge an assertive diagnosis in many cases. Reda and colleagues (2013) report warning signs of IEI, which focus on the site, severity, and frequency of recurrent infection as best practices to guide the study of IEI [25, 26].

By employing a genomic-based strategy, candidate disease-causing variants associated with nine different genetic disorders were detected in 13 subjects. Seven disorders were previously classified as IEI, being (i) predominantly antibody deficiencies due to variants in the *BTK* gene, the most abundant disorders in the cohort. Two genes associated with (ii) immunodeficiencies affecting cellular and humoral immunity were identified. The remaining IEI disorders were related to (iii) combined immunodeficiencies with associated or syndromic features, (iv) congenital defects of phagocyte number or function, (v) complement deficiencies, and (vi) regulatory T cell defect. The WES analysis also identified four variants in two genes related to (vii) non-IEI disorders, associated with rare skin diseases, metabolic and blood disorders. Though the disorders were not included in the list of IEI-related diseases, the phenotypes observed in the patients are similar to other genetically determined IEI disorders.

Five different SNVs were identified in the *BTK* gene related to X-Linked Agammaglobulinemia (XLA; OMIM #300300) representing the most prevalent disorder in the cohort. Predominantly antibody deficiencies have been reported as the most prevalent IEI disorders reaching approximately 50% of the cases globally reported [27, 28]. XLA is typically characterized by very low immunoglobulin serum levels and the absence of peripheral B cells due to its arrest at the pre-B or mature B cells differentiation stages [29, 30]. Interestingly, other deleterious variants were reported in some residues altered in the patients, indicating the relevance of such regions in the XLA pathogenesis [31–34]. The nonsense variant (p.Tyr112*) harbored by patient 4 is a well-characterized SNV that promotes a premature stop-codon gain at the PH domain [31]. This residue is crucial to maintain the stability and function of BTK protein [31, 35].

Combined B and T cell defects accounted for two (*CD40LG* and *CARD11*) out of the seven IEI-related genes in this analysis. This group of disorders includes T cell-negative and B cell-positive severe combined immunodeficiencies (SCID), T cell-negative and B cell-negative SCID; and combined immunodeficiencies (CID), generally less severe than SCID [5]. The disorders reported in the genes identified in this group were classified as CID. An exome-wide search was crucial to establish the genetic diagnosis in patient 6 given his agammaglobulinemia phenotype with hyper-IgM findings. One case of X-Linked Hyper IgM Syndrome, attributed to an in-frame deletion (p.Tyr146del) in the *CD40LG* gene, was reported. Clinical manifestations like this patient may misinterpret an XLA diagnosis [36]. The p.Tyr146del was also reported in a Vietnamese patient with X‐linked hyper‐IgM syndrome [37]. Clinical findings also pointed toward the X-linked inheritance with the patients showing early death of male family members [36, 38, 39].

Within the CID disorders group, the missense variant (p.Leu251Pro) in the *CARD11* gene classified as likely pathogenic in this analysis displayed a conflict of interpretations about the variant pathogenicity in ClinVar. Experimental analyses showed that variants in the *CARD11* gene were associated with aberrant growth and survival of lymphocytes allowing lead to both malignancy and monogenic primary immunodeficiencies [40–44]. The p.Leu251Pro causes a gain of function (GOF) in the *CARD11*, driving an in vitro cell proliferation and a constitutive NF-κB activation, which is required for lymphocyte activation, survival, and proliferation during the adaptive immune response [45]. Notably, the reported phenotype matches the lymphoproliferation and B lymphocytosis observed in patient 7.

Although some categories of IEI were less frequent in the studied cohort, the variants reported by WES analysis provided valuable insight into the genetic diagnosis of the patients. For example, the Wiskott–Aldrich Syndrome (WAS; OMIM #301000) reported in patient 8 caused by the likely pathogenic nonsense variant (p.Gln297Ter) is a rare genetic illness occurring between 1–10 males per million [46]. WAS is a CID with associated or syndromic features inherited under an X-linked recessive model. The disorder is associated with thrombocytopenia, eczema, and a remarkably high percentage of autoimmunity disease [46–48]. The p.Gln297Ter relies on the P21-Rho-binding domain responsible for binding a highly conserved GTPase domain that controls cellular morphogenesis [49–51]. Unlike the previous report SNVs, the alteration detected in the *CYBB* gene showed a synonymous effect (p.Lys161 =), has been classified as pathogenic by ACMG rules and Likely pathogenic by ClinVar, and is associated with a pathogenic outcome previously reported in a patient with chronic granulomatous disease (CGD) and myelodysplastic syndrome [52]. The harmful potential of the p.Lys161 = variant has been studied, evidencing alteration of the broken donor site, which affects splicing and leads to the exon skipping event. It disrupts *CYBB* gene function and protein formation [52]. The exon skipping event on the *CYBB* gene is reported as one of the causes of CGD phenotype [53, 54]. CGD is a primary phagocytic deficiency resulting in defects of respiratory burst [5]. Variants in *CYBB* account for more than 60% of all CGD cases, with about 10–15% of *CYBB*-related CGD being caused by new germline variants [55, 56].

Complement deficiency was represented by a pathogenic frameshift variant (p.Gln380SerfsTer7) in the *C6* gene, causing C6 deficiency (OMIM #612446). Usually, low-throughput genetic investigations focused on target amplification of the exonic regions that commonly accumulate most pathogenic deletions, such as exons 6, 7 and 12 of the *C6* gene [57, 58]. The reported indel was in exon 8 of *C6*, corroborating the breadth of WES analysis [57, 58]. Finally, the last category of IEI-related disorders found was defects in intrinsic and innate immunity. Two heterozygous pathogenic variants in the *LRBA* gene suggest a compound heterozygous genotype that leads to immunodeficiency, common variable, 8, with autoimmunity (OMIM #614700), which confirm the genotype–phenotype correlation, since patient 11 presented several disorders resulting from autoimmune manifestation as diabetes mellitus, pancytopenia/bicytopenia, hypothyroidism and celiac disease [59–61].

Two subjects (patients 12 and 13) initially enrolled in the study with symptoms overlapping candidate IEI disorders were found to harbor deleterious variants in genes other than those known to cause IEI. Both patients met the clinical inclusion criteria since the IEI has heterogeneous phenotypic features related to overlapping clinical manifestations and misdiagnosis [3, 6, 62]. In *ABCA12*, the heterozygous likely pathogenic splicing variant (c.318-2A > G) and a VUS missense (p.Asn678Ser) SNV might be involved in Ichthyosis, congenital, autosomal recessive 4B (harlequin) (OMIM #242500) in patient 12. Compound heterozygous (CH) genotypes have also been detected as a possible reason for the less severe congenital ichthyosis [63–66]. Akiyama and colleagues (2010) reported several variant combinations that result in compound heterozygosity and the disease outcome. This study suggests combining nonsense, frameshift, or splice site variants with a second missense variant leads to a high prevalence of harlequin ichthyosis phenotype [63–66]. The identification of VUS usually leads to the discovery of novel variants that, with a genotype–phenotype correlation, can help in the understanding of this heterogeneous disorder [67]. Although this occurrence reveals novel Ichthyosis-causing variants, additional investigation, such as computational pathogenicity prediction, family history, and laboratory validation are needed to determine these VUS as responsible for the clinical phenotype [68].

Heterozygous likely pathogenic variants suggest that compound heterozygosity genotype was also identified in the *SLC25A13* gene. The p.Arg585His was reported with a CH genotype in a Chinese female patient with neonatal intrahepatic cholestasis caused by citrin deficiency (NICCD), also known as Citrullinemia, type II, neonatal-onset (OMIM #605814) [69]. Patient 13, p.Arg585His was observed with a second likely pathogenic variant, p.Pro540Ser. NICCD leads to suppression of the bile flow, usually not severe [69].

The patients described in this study often originated from low-income Brazilian families and were treated by the national public health system. The limited availability for performing some immunological tests, discontinuity in the patient follow-up and absence of access to clinical data challenge an in-depth correlation between the genetic background and the phenotype of each patient. Similar limitations are faced with validating the functional effects and the presence of the variants by alternative molecular biology methods. Combining WES findings and functional analyses allows a better resolution of genotype–phenotype correlation, improving the variant classification and the discovery of new disease-associated genes [7, 10–13, 70]. Though it was not possible to reach such a level of evidence in the analysis of this study, considering that most of the patients had a previous history of unknown death among relatives, the findings reported in the present research were crucial to providing insights into the genetic source of their phenotype. The match observed between the patient's phenotype and the SNVs harbored by them should be further investigated in future studies. Though additional cases harboring the variants firstly reported here might reinforce the genotype–phenotype correlation observed, caution was exercised to discourage over-interpretation of the data for guiding clinical decisions.

## Conclusions

Diagnosing highly heterogeneous genetic disorders, such as inborn errors of immunity (IEI), poses significant challenges in identifying disease-causing variants. This is particularly true in low-income countries like Brazil, where access to genetic sequencing is especially difficult. The recent advances in sequencing technologies have shown promise in overcoming these limitations and providing a comprehensive overview of the genetic landscape, even within resource-constrained settings. In this study conducted among patients treated by the Brazilian public health system, whole exome sequencing (WES) was utilized to improve the genetic characterization of IEI disorders significantly. This approach holds immense potential for implementation within the Brazilian public Unified Health System, offering a transformative impact on patient care and management. The present analysis revealed genetic variants in genes associated with six distinct categories of IEI disorders, confirming the clinical manifestations observed in all patients.

Moving forward, it is crucial to recognize the perspectives and challenges in implementing genetic sequencing analysis within the context of Brazil's public Unified Health System. By overcoming barriers such as cost, infrastructure, and access to specialized expertise, there is an opportunity to extend the benefits of genetic screening to a broader patient population, ensuring equitable healthcare services. This would enhance the genetic diagnosis of IEI disorders and facilitate the development of tailored treatment approaches and genetic counseling services. Nevertheless, additional studies are required to delve deeper into the molecular mechanisms underlying the alterations caused by the identified SNVs in the genetic screening. Such investigations will contribute to a more comprehensive understanding of the pathogenesis of IEI disorders and further refine clinical management strategies. In conclusion, the findings highlight the transformative potential of implementing WES within the Brazilian public health system for advancing the diagnosis and management of IEI disorders. Addressing the challenges specific to low-income countries like Brazil can pave the way for integrating genetic sequencing technologies into routine clinical practice, ultimately improving patient outcomes and fostering a more inclusive and comprehensive healthcare system.

## Materials and methods

### Setting

A genetic screening was conducted in 13 unrelated patients with suspicion of IEI treated by the Brazilian public health system across different medical centers in Rio de Janeiro between 2017 and 2018. Patients were being followed up by medical centers awaiting genetic counseling to aid in the diagnosis. All diagnosed IEIs were classified according to the Primary Immunodeficiency Classification of the International Union of Immunological Societies (IUIS) Expert Committee, updated in 2022 [5]. The inclusion criteria considered the ten IEI warning signs promoted by the Jeffrey Modell Foundation: ≥ 4 ear infections in one year; ≥ 2 serious sinus infections in one year; ≥ 2 months on antibiotics with little effect; ≥ 2 cases of pneumonias in one year; Failure of an infant to gain weight or grow normally; Recurrent, deep skin or organ abscesses; Persistent thrush in mouth or fungal; Infection on the skin; Need for intravenous antibiotics to clear infections; ≥ 2 deep-seated infections including septicemia; Family history of IEI [27, 71–73]. Additional warning signs were considered as listed by Pinto-Mariz and Goudouris (2021) [28]. The studied cohort included five patients admitted to the Instituto de Puericultura e Pediatria Martagão Gesteira (IPPMG) of the Universidade Federal do Rio de Janeiro (UFRJ), seven from the Serviço de Alergia e Imunologia, do Instituto Fernandes Figueira (IFF) in the Fundação Oswaldo Cruz (FIOCRUZ), and one from Allergy and Immunology Sector of the Pediatric Service of the Federal Hospital of Rio de Janeiro State (HFSE)—Ministry of Health. A peripheral venous blood sample was taken from each patient, along with their clinical history and relevant laboratory results. All subjects and their guardians agreed to participate in this study by signing a written informed consent. The Institutional Ethical Committee approved the Instituto Fernandes Figueira study protocol (no. CAAE42934815.4.0000.52695269), and the Ethical Committee of the Instituto Nacional do Câncer (153/10).

### DNA extraction, whole exome sequencing, and variant calling framework

Genomic DNA was extracted from each patient's peripheral blood lymphocytes using the QIAmp DNA Mini Kit® (QIAGEN®) according to the manufacturer’s instructions. The WES libraries were prepared using Illumina TruSeq® Exome Kit (8 rxn × 6plex) according to the manufacturer's protocol. The Illumina NextSeq® 500/550 High Output Kit v2 (150 cycles) was used, yielding 2 × 75 bp paired-end reads to generate the sequencing data. The raw data files in FASTQ format were processed in an in-house bioinformatic pipeline described by us [74–77]. The computational framework used includes reads mapping, quality control, and variant calling and annotation. The fastqc (http://www.bioinformatics.babraham.ac.uk/projects/fastqc/) and Trimmomatic [78] were used to inspect the quality of sequences generated and remove bad-formed reads. The remaining sequences were mapped to the human reference genome (GRCh38) using Bowtie2 version 2.3.5.1 [79, 80]. Additional BAM file manipulations were performed with Samtools version 1.11 [81] for sorting and mapping quality filtration (Q30). Duplicate reads were marked using Picard MarkDuplicates tool version 2.20.7 (http://broadinstitute.github.io/picard). Using Genome Analysis Toolkit (GATK) software version 4.1.20 [82], to recalibration of the base quality of BAM files was used Base Quality Score Recalibration (BQSR) steps followed by germline variant calling in the HaplotypeCaller tool. To annotate the genetic consequences, populational allele frequencies, molecular impact, and effects of the variants identified, was used version 5.0 of SnpEff and SnpSift software [83, 84]. Tablet graphical viewer software was used to visualize the read mappings from BAM files [85].

### Filtering potential harmful single nucleotide variants

A list of 585 IEI-related genes panel, as previously described in the literature, was utilized to select potential pathogenic variants [5, 27, 86–90]. The analysis was focused on germline and rare (minor frequency allele ≤ 0.01) protein-altering variants, including truncating variants (stop gain/loss, start loss, or frameshift), missense variants, canonical splice-site variants, in-frame insertions, deletions, and indels. Additionally was integrated prediction scores of pathogenicity from different computational tools such as SIFT (Sorting Intolerant from Tolerant) [91], PolyPhen (Polymorphism Phenotyping) [92], CADD (Combined Annotation-Dependent Depletion) [93], and LoFtool [94] according to the Ensembl Variant Effect Predictor database (VEP) [95]. The variant classification strategy was based on the American College of Medical Genetics and Genomics and the Association for Molecular Pathology (ACMG/AMP) guidelines [96]. Two approaches to select qualifying variants. First, the VarSome clinical database [97] was employed to prioritize germline pathogenic and likely pathogenic variants based on ACMG guidelines. Secondly, the Franklin (http://franklin.genoox.com) tool was used for phenotype-based variants prioritization according to Human Phenotype Ontology (HPO) terms. Additionally, a target gene investigation was performed considering the panel for primary Immunodeficiency Classification of the International Union of Immunological Societies (IUIS) Expert Committee, updated in 2022 [5]. The annotation of inheritance pattern was considered the Online Mendelian Inheritance in Man (OMIM) database and the population frequency of the variants was researched in 1000 Genomes Project, Exome Aggregation Consortium (ExAC) and Genome Aggregation Database (gnomAD) [98–102]. The filtering approach is shown in Supplementary Figure S3.

### Downstream genetic variant analysis for advancing Genotype–Phenotype correlation

The validation of homozygous variant NM_001115131.3:c.1138del (p.Gln380SerfsTer7) in patient 10, was done with the polymerase chain reaction (PCR) combined with bidirectional Sanger sequencing. Primers targeting the variant site were designed for PCR-amplification (ThermoFisher Cycler, Waltham, MA, USA) and sequencing (Forward Primer: 5'-GATTCTAGTTTTATTAGGAT-3' and Reverse Primer: 5'-AAAAATGTATTGCATGCTAT-3'). Primers and PCR products were purified using PureLink® Invitrogen™ (Thermo Fisher Scientific, Waltham, MA, USA) and on an automated sequencer ABI 3730 Genetic analyzer (Thermo Fisher Scientific, Waltham, MA, USA). The results were interpreted by the software BioEdit.

In silico analysis was performed to investigate the splicing regions of synonymous variant NM_000397.4:c.483G > A (p.Lys161 =) in the *CYBB* gene found in patient 9. The tools for predicting splicing defects have the potential to aid in disease diagnosis by facilitating a deeper understanding of the splicing mechanism [103]. The prediction analysis was performed using NetGene2; Alternative Splice Site Predictor (ASSP); VEP splicing prediction (Ada score, RF score, and MaxEntScan); ESEfinder; and NNSplice tools with corresponding prediction score thresholds and sequence lengths to reach a sensitivity, and specificity ≥ 80% [95, 104–108].

To confirm the phase of the variants in genes *LRBA*, *ABCA12* and *SLC25A13*, read-based phasing analysis was conducted using HapCUT2 with default parameters accessing germline WES BAM files and respective VCF files [109]. Given that the analysis focused on the reconstruction of haplotype blocks with increased resolution directly proportional to the distance of the variations in the gene, the size of the reads on the Illumina platform (100–250 bases) must be considered [109].

### Supplementary Information

**Additional file 1:** **Figure S1.**

**Additional file 2:** **Figure S2.**

**Additional file 3:** **Figure S3.**

**Additional file 4:** **Table S1.**

**Additional file 5:** **Table S2.**

**Additional file 6:** **Table S3.**

**Additional file 7:** **Table S4.**

## Data Availability

The WES dataset used in this study is publicly available in SRA-NCBI, SRA accession PRJNA899588 (https://www.ncbi.nlm.nih.gov/bioproject/PRJNA899588).

## Abbreviations

ACMG/AMP: American College of Medical Genetics and Genomics and the Association for Molecular Pathology
AD: Autosomal dominant
AR: Autosomal recessive
ASSP: Alternative Splice Site Predictor
BQSR: Base Quality Score Recalibration
CADD: Combined Annotation-Dependent Depletion
CGD: Chronic granulomatous disease
CH: Compound heterozygous
CID: Combined Immunodeficiency
ExAC: Exome Aggregation Consortium
FIOCRUZ: Fundação Oswaldo Cruz
GATK: Genome Analysis Toolkit
gnomAD: Genome Aggregation Database
GOF: Gain of function
G6PD: Glucose-6-phosphate dehydrogenase deficiency
HFSE: Hospital Federal dos servidores do Estado
HPO: Human Phenotype Ontology
IEI: Inborn error of Immunity
IFF: Instituto Fernandes Figueira
IPPMG: Instituto de Puericultura e Pediatria Martagão Gesteira
IUIS: International Union of Immunological Societies
NGS: Next Generation Sequencing
NICCD: Neonatal intrahepatic cholestasis caused by citrin deficiency
OMIM: Online Mendelian Inheritance in Man
PCR: Polymerase chain reaction
P/LP: Pathogenic/Likely pathogenic
PH: N-terminal pleckstrin homology
PID: Primary immunodeficiency
PolyPhen: Polymorphism Phenotyping
SCID: Severe combined immunodeficiencies
SH2: SRC homology 2
SH3: SRC homology 3
SIFT: Sorting Intolerant from Tolerant
SNV: Single nucleotide Variant
SUS: Sistema Único de Saúde
TH: TEC homology
TK: Tyrosine Kinase
UFRJ: Universidade Federal do Rio de Janeiro
VCF: Variant call format
VEP: Variant Effect Predictor database
VUS: Variant of uncertain Significance
WAS: Wiskott–Aldrich Syndrome
WES: Whole exome sequencing
WGS: Whole genome sequencing
XLA: X-linked Agammaglobulinemia
XLR: X-Linked recessive
y.o.: Years old

## References

[1] Tangye SG, Al-Herz W, Bousfiha A, Chatila T, Cunningham-Rundles C, Etzioni A (2020). Human inborn errors of immunity: 2019 update on the classification from the International Union of Immunological Societies Expert Committee. J Clin Immunol.

[2] Li PH, Wong WW, Leung EN, Lau C-S, Au E. Novel pathogenic mutations identified in the first Chinese pedigree of complete C6 deficiency. Clin Transl Immunology 2020;9:e1148. 10.1002/cti2.1148. 10.1002/cti2.1148 PMC7343556 32670577

[3] Notarangelo LD, Bacchetta R, Casanova J-L, Su HC. Human inborn errors of immunity: An expanding universe. Sci Immunol 2020;5. 10.1126/sciimmunol.abb1662. 10.1126/sciimmunol.abb1662 PMC7647049 32651211

[4] Condino-Neto A (2014). The relevance of collaborative work: the Latin American Society for Immunodeficiencies (LASID) registry model. Clin Exp Immunol.

[5] Tangye SG, Al-Herz W, Bousfiha A, Cunningham-Rundles C, Franco JL, Holland SM (2022). Human inborn errors of immunity: 2022 update on the classification from the International Union of Immunological Societies Expert Committee. J Clin Immunol.

[6] Delmonte OM, Castagnoli R, Calzoni E, Notarangelo LD (2019). Inborn errors of immunity with immune dysregulation: from bench to bedside. Front Pediatr.

[7] Engelbrecht C, Urban M, Schoeman M, Paarwater B, van Coller A, Abraham DR, et al. Clinical utility of whole exome sequencing and targeted panels for the identification of inborn errors of immunity in a resource-constrained setting. Front Immunol 2021;12:665621. 10.3389/fimmu.2021.665621. 10.3389/fimmu.2021.665621 PMC8176954 34093558

[8] Griffith LM, Cowan MJ, Notarangelo LD, Kohn DB, Puck JM, Pai S-Y (2014). Primary Immune Deficiency Treatment Consortium (PIDTC) report. J Allergy Clin Immunol.

[9] Raje N, Soden S, Swanson D, Ciaccio CE, Kingsmore SF, Dinwiddie DL (2014). Utility of next generation sequencing in clinical primary immunodeficiencies. Curr Allergy Asthma Rep.

[10] Zhang Y, Su HC, Lenardo MJ (2015). Genomics is rapidly advancing precision medicine for immunological disorders. Nat Immunol.

[11] Arts P, Simons A, AlZahrani MS, Yilmaz E, AlIdrissi E, van Aerde KJ (2019). Exome sequencing in routine diagnostics: a generic test for 254 patients with primary immunodeficiencies. Genome Med.

[12] Cifaldi C, Brigida I, Barzaghi F, Zoccolillo M, Ferradini V, Petricone D (2019). Targeted NGS platforms for genetic screening and gene discovery in primary immunodeficiencies. Front Immunol.

[13] Yska HAF, Elsink K, Kuijpers TW, Frederix GWJ, van Gijn ME, van Montfrans JM (2019). Diagnostic yield of next generation sequencing in genetically undiagnosed patients with primary immunodeficiencies: a systematic review. J Clin Immunol.

[14] Stranneheim H, Wedell A (2016). Exome and genome sequencing: a revolution for the discovery and diagnosis of monogenic disorders. J Intern Med.

[15] Nijman IJ, van Montfrans JM, Hoogstraat M, Boes ML, van de Corput L, Renner ED (2014). Targeted next-generation sequencing: a novel diagnostic tool for primary immunodeficiencies. J Allergy Clin Immunol.

[16] Biesecker LG, Shianna KV, Mullikin JC (2011). Exome sequencing: the expert view. Genome Biol.

[17] Chou J, Ohsumi TK, Geha RS (2012). Use of whole exome and genome sequencing in the identification of genetic causes of primary immunodeficiencies. Curr Opin Allergy Clin Immunol.

[18] Barbitoff YA, Polev DE, Glotov AS, Serebryakova EA, Shcherbakova IV, Kiselev AM (2020). Systematic dissection of biases in whole-exome and whole-genome sequencing reveals major determinants of coding sequence coverage. Sci Rep.

[19] Roskoski R (2016). Ibrutinib inhibition of Bruton protein-tyrosine kinase (BTK) in the treatment of B cell neoplasms. Pharmacol Res.

[20] Agnew C, Jura N (2017). Switching on BTK-One Domain at a Time. Structure.

[21] de Melo KM, Alves LM, Valente CFC, Tavares FS (2022). One-year intravenous immunoglobulin replacement therapy: efficacy in reducing hospital admissions in pediatric patients with Inborn Errors of Immunity. J Pediatr.

[22] Suspitsin EN, Guseva MN, Kostik MM, Sokolenko AP, Skripchenko NV, Levina AS (2020). Next generation sequencing analysis of consecutive Russian patients with clinical suspicion of inborn errors of immunity. Clin Genet.

[23] Jouanguy E, Béziat V, Mogensen TH, Casanova J-L, Tangye SG, Zhang S-Y (2020). Human inborn errors of immunity to herpes viruses. Curr Opin Immunol.

[24] Barreto ICDP, Barreto BAP, Cavalcante EG do N, Condino Neto A. Immunological deficiencies: more frequent than they seem to be. J Pediatr 2021;97 Suppl 1:S49–58. 10.1016/j.jped.2020.10.009. 10.1016/j.jped.2020.10.009 PMC9432333 33238140

[25] Subbarayan A, Colarusso G, Hughes SM, Gennery AR, Slatter M, Cant AJ (2011). Clinical features that identify children with primary immunodeficiency diseases. Pediatrics.

[26] Reda SM, El-Ghoneimy DH, Afifi HM (2013). Clinical predictors of primary immunodeficiency diseases in children. Allergy Asthma Immunol Res.

[27] Modell V, Orange JS, Quinn J, Modell F (2018). Global report on primary immunodeficiencies: 2018 update from the Jeffrey Modell Centers Network on disease classification, regional trends, treatment modalities, and physician reported outcomes. Immunol Res.

[28] Pinto-Mariz F, Goudouris E. Inborn errors of immunity: What to look for beyond infections. J Immunol Sci. 2021;5.

[29] Smith T, Cunningham-Rundles C (2019). Primary B-cell immunodeficiencies. Hum Immunol.

[30] Suri D, Rawat A, Singh S (2016). X-linked Agammaglobulinemia. Indian J Pediatr.

[31] Fiorini M, Franceschini R, Soresina A, Schumacher R-F, Ugazio AG, Rossi P (2004). BTK: 22 novel and 25 recurrent mutations in European patients with X-linked agammaglobulinemia. Hum Mutat.

[32] Holinski-Feder E, Weiss M, Brandau O, Jedele KB, Nore B, Bäckesjö CM (1998). Mutation screening of the BTK gene in 56 families with X-linked agammaglobulinemia (XLA): 47 unique mutations without correlation to clinical course. Pediatrics.

[33] López-Granados E, Pérez de Diego R, Ferreira Cerdán A, Fontán Casariego G, García Rodríguez MC. A genotype-phenotype correlation study in a group of 54 patients with X-linked agammaglobulinemia. J Allergy Clin Immunol 2005;116:690–7. 10.1016/j.jaci.2005.04.043. 10.1016/j.jaci.2005.04.043 16159644

[34] Tóth B, Volokha A, Mihas A, Pac M, Bernatowska E, Kondratenko I (2009). Genetic and demographic features of X-linked agammaglobulinemia in Eastern and Central Europe: a cohort study. Mol Immunol.

[35] Velickovic M, Prasad ML, Weston SA, Benson EM (2004). Identification of the bruton tyrosine kinase (BTK) gene mutations in 20 Australian families with X-linked agammaglobulinemia (XLA). Hum Mutat.

[36] Smith Z, Przebinda A, Zia H, Khalid B, Cherry M, Guild R. Hyper IgM Masquerading as Bruton’s Agammaglobulinemia: a case report: 1374. ACG. 2017;112:S744.

[37] Phan ANL, Pham TTT, Phan XT, Huynh N, Nguyen TM, Cao CTT, et al. CD40LG mutations in Vietnamese patients with X-linked hyper-IgM syndrome; catastrophic anti-phospholipid syndrome as a new complication. Mol Genet Genomic Med 2021;9:e1732. 10.1002/mgg3.1732. 10.1002/mgg3.1732 PMC8404229 34114358

[38] Wang L-L, Zhou W, Zhao W, Tian Z-Q, Wang W-F, Wang X-F, et al. Clinical features and genetic analysis of 20 Chinese patients with X-linked hyper-IgM syndrome. J Immunol Res 2014;2014:683160. 10.1155/2014/683160. 10.1155/2014/683160 PMC4158165 25215306

[39] Winkelstein JA, Marino MC, Ochs H, Fuleihan R, Scholl PR, Geha R (2003). The X-linked hyper-IgM syndrome: clinical and immunologic features of 79 patients. Medicine.

[40] Turvey SE, Durandy A, Fischer A, Fung S-Y, Geha RS, Gewies A (2014). The CARD11-BCL10-MALT1 (CBM) signalosome complex: Stepping into the limelight of human primary immunodeficiency. J Allergy Clin Immunol.

[41] Jones TA, Hutcherson SM, Bedsaul JR, Pomerantz JL. Dysregulated CARD11 signaling in the development of diffuse large B cell lymphoma. LymphoSign J 2020;7:90–103. 10.14785/lymphosign-2020-0006.

[42] Lenz G, Davis RE, Ngo VN, Lam L, George TC, Wright GW (2008). Oncogenic CARD11 mutations in human diffuse large B cell lymphoma. Science.

[43] Lamason RL, McCully RR, Lew SM, Pomerantz JL (2010). Oncogenic CARD11 mutations induce hyperactive signaling by disrupting autoinhibition by the PKC-responsive inhibitory domain. Biochemistry.

[44] Chan W, Schaffer TB, Pomerantz JL (2013). A quantitative signaling screen identifies CARD11 mutations in the CARD and LATCH domains that induce Bcl10 ubiquitination and human lymphoma cell survival. Mol Cell Biol.

[45] Pedersen SM, Chan W, Jattani RP, Mackie DS, Pomerantz JL (2015). Negative Regulation of CARD11 Signaling and Lymphoma Cell Survival by the E3 Ubiquitin Ligase RNF181. Mol Cell Biol.

[46] Cleland SY, Siegel RM (2011). Wiskott-Aldrich Syndrome at the nexus of autoimmune and primary immunodeficiency diseases. FEBS Lett.

[47] Dupuis-Girod S, Medioni J, Haddad E, Quartier P, Cavazzana-Calvo M, Le Deist F (2003). Autoimmunity in Wiskott-Aldrich syndrome: risk factors, clinical features, and outcome in a single-center cohort of 55 patients. Pediatrics.

[48] Albert MH, Bittner TC, Nonoyama S, Notarangelo LD, Burns S, Imai K (2010). X-linked thrombocytopenia (XLT) due to WAS mutations: clinical characteristics, long-term outcome, and treatment options. Blood.

[49] Miller PJ, Johnson DI (1994). Cdc42p GTPase is involved in controlling polarized cell growth in Schizosaccharomyces pombe. Mol Cell Biol.

[50] Ochs HD, Thrasher AJ. The Wiskott-Aldrich syndrome. J Allergy Clin Immunol 2006;117:725–38; quiz 739. 10.1016/j.jaci.2006.02.005. 10.1016/j.jaci.2006.02.005 16630926

[51] Blum M, Chang H-Y, Chuguransky S, Grego T, Kandasaamy S, Mitchell A (2021). The InterPro protein families and domains database: 20 years on. Nucleic Acids Res.

[52] Reis BCS, Cunha DP, Bueno APS, Carvalho FAA, Dutra J, Mello FV, et al. Chronic Granulomatous Disease and Myelodysplastic Syndrome in a Patient with a Novel Mutation in CYBB. Genes 2021;12. 10.3390/genes12101476. 10.3390/genes12101476 PMC8535487 34680870

[53] de Boer M, van Leeuwen K, Hauri-Hohl M, Roos D. Activation of cryptic splice sites in three patients with chronic granulomatous disease. Mol Genet Genomic Med 2019;7:e854. 10.1002/mgg3.854. 10.1002/mgg3.854 PMC6732321 31364312

[54] de Boer M, van Leeuwen K, Geissler J, Belohradsky BH, Kuijpers TW, Roos D (2017). Mutation in an exonic splicing enhancer site causing chronic granulomatous disease. Blood Cells Mol Dis.

[55] Rider NL, Jameson MB, Creech CB (2018). Chronic granulomatous disease: epidemiology, pathophysiology, and genetic basis of disease. J Pediatric Infect Dis Soc.

[56] Mollin M, Beaumel S, Vigne B, Brault J, Roux-Buisson N, Rendu J (2021). Clinical, functional and genetic characterization of 16 patients suffering from chronic granulomatous disease variants - identification of 11 novel mutations in CYBB. Clin Exp Immunol.

[57] Dragon-Durey MA, Fremeaux-Bacchi V, Blouin J, Barraud D, Fridman WH, Kazatchkine MD (2003). Restricted genetic defects underlie human complement C6 deficiency. Clin Exp Immunol.

[58] Parham KL, Roberts A, Thomas A, Würzner R, Henderson HE, Potter PC (2007). Prevalence of mutations leading to complete C6 deficiency (C6Q0) in the Western Cape, South Africa and detection of novel mutations leading to C6Q0 in an Irish family. Mol Immunol.

[59] Galati A, Muciaccia R, Marucci A, Di Paola R, Menzaghi C, Ortolani F, et al. Early-Onset Diabetes in an Infant with a Novel Frameshift Mutation in LRBA. Int J Environ Res Public Health 2022;19. 10.3390/ijerph191711031. 10.3390/ijerph191711031 PMC9517908 36078750

[60] Sari S, Dogu F, Hwa V, Haskologlu S, Dauber A, Rosenfeld R (2016). A successful HSCT in a girl with novel LRBA mutation with refractory celiac disease. J Clin Immunol.

[61] Johnson MB, De Franco E, Lango Allen H, Al Senani A, Elbarbary N, Siklar Z (2017). Recessively inherited LRBA mutations cause autoimmunity presenting as neonatal diabetes. Diabetes.

[62] Tengsujaritkul M, Suratannon N, Ittiwut C, Ittiwut R, Chatchatee P, Suphapeetiporn K, et al. Phenotypic heterogeneity and genotypic spectrum of inborn errors of immunity identified through whole exome sequencing in a Thai patient cohort. Pediatr Allergy Immunol 2022;33:e13701. 10.1111/pai.13701. 10.1111/pai.13701 34796988

[63] Akiyama M, Sakai K, Sugiyama-Nakagiri Y, Yamanaka Y, McMillan JR, Sawamura D (2006). Compound heterozygous mutations including a de novo missense mutation in ABCA12 led to a case of harlequin ichthyosis with moderate clinical severity. J Invest Dermatol.

[64] Shibata A, Sugiura K, Suzuki A, Ichiki T, Akiyama M (2015). Apparent homozygosity due to compound heterozygosity of one point mutation and an overlapping exon deletion mutation in ABCA12: a genetic diagnostic pitfall. J Dermatol Sci.

[65] Kelsell DP, Norgett EE, Unsworth H, Teh M-T, Cullup T, Mein CA (2005). Mutations in ABCA12 underlie the severe congenital skin disease harlequin ichthyosis. Am J Hum Genet.

[66] Akiyama M (2010). ABCA12 mutations and autosomal recessive congenital ichthyosis: a review of genotype/phenotype correlations and of pathogenetic concepts. Hum Mutat.

[67] Almarzooqi F, Souid A-K, Vijayan R, Al-Hammadi S. Novel genetic variants of inborn errors of immunity. PLoS One 2021;16:e0245888. 10.1371/journal.pone.0245888. 10.1371/journal.pone.0245888 PMC7822508 33481921

[68] Zama D, Conti F, Moratti M, Cantarini ME, Facchini E, Rivalta B (2021). Immune cytopenias as a continuum in inborn errors of immunity: An in-depth clinical and immunological exploration. Immun Inflamm Dis.

[69] Fu H-Y, Zhang S-R, Wang X-H, Saheki T, Kobayashi K, Wang J-S (2011). The mutation spectrum of the SLC25A13 gene in Chinese infants with intrahepatic cholestasis and aminoacidemia. J Gastroenterol.

[70] Thaventhiran JED, Lango Allen H, Burren OS, Rae W, Greene D, Staples E (2020). Whole-genome sequencing of a sporadic primary immunodeficiency cohort. Nature.

[71] Arkwright PD, Gennery AR (2011). Ten warning signs of primary immunodeficiency: a new paradigm is needed for the 21st century. Ann N Y Acad Sci.

[72] Costa-Carvalho BT, Grumach AS, Franco JL, Espinosa-Rosales FJ, Leiva LE, King A (2014). Attending to warning signs of primary immunodeficiency diseases across the range of clinical practice. J Clin Immunol.

[73] Condino-Neto A, Sorensen RU, Gómez Raccio AC, King A, Espinosa-Rosales FJ, Franco JL (2015). Current state and future perspectives of the Latin American Society for Immunodeficiencies (LASID). Allergol Immunopathol.

[74] Francisco Junior R da S, de Morais GL, de Carvalho JB, Dos Santos Ferreira C, Gerber AL, de C Guimarães AP, et al. Clinical and genetic findings in two siblings with X-Linked agammaglobulinemia and bronchiolitis obliterans: a case report. BMC Pediatr 2022;22:181. 10.1186/s12887-022-03245-x. 10.1186/s12887-022-03245-x PMC8981605 35382780

[75] Borda V, da Silva Francisco Junior R, Carvalho JB, Morais GL, Duque Rossi Á, Pezzuto P, et al. Whole-exome sequencing reveals insights into genetic susceptibility to Congenital Zika Syndrome. PLoS Negl Trop Dis. 2021;15:e0009507. 10.1371/journal.pntd.0009507. 10.1371/journal.pntd.0009507 PMC8224898 34125832

[76] Aguiar RS, Pohl F, Morais GL, Nogueira FCS, Carvalho JB, Guida L, et al. Molecular alterations in the extracellular matrix in the brains of newborns with congenital Zika syndrome. Sci Signal 2020;13. 10.1126/scisignal.aay6736. 10.1126/scisignal.aay6736 32518143

[77] Alves-Leon SV, Ferreira CDS, Herlinger AL, Fontes-Dantas FL, Rueda-Lopes FC, Francisco R da S Jr, et al. Exome-wide search for genes associated with central nervous system inflammatory demyelinating diseases following CHIKV infection: the tip of the iceberg. Front Genet. 2021;12:639364. 10.3389/fgene.2021.639364. 10.3389/fgene.2021.639364 PMC8010313 33815474

[78] Bolger AM, Lohse M, Usadel B (2014). Trimmomatic: a flexible trimmer for Illumina sequence data. Bioinformatics.

[79] Langmead B, Salzberg SL (2012). Fast gapped-read alignment with Bowtie 2. Nat Methods.

[80] Langmead B, Wilks C, Antonescu V, Charles R (2019). Scaling read aligners to hundreds of threads on general-purpose processors. Bioinformatics.

[81] Li H, Handsaker B, Wysoker A, Fennell T, Ruan J, Homer N (2009). The sequence alignment/map format and SAMtools. Bioinformatics.

[82] McKenna A, Hanna M, Banks E, Sivachenko A, Cibulskis K, Kernytsky A (2010). The genome analysis toolkit: a MapReduce framework for analyzing next-generation DNA sequencing data. Genome Res.

[83] Cingolani P, Patel VM, Coon M, Nguyen T, Land SJ, Ruden DM (2012). Using drosophila melanogaster as a model for genotoxic chemical mutational studies with a new program. SnpSift Front Genet.

[84] Cingolani P, Platts A, Wang LL, Coon M, Nguyen T, Wang L (2012). A program for annotating and predicting the effects of single nucleotide polymorphisms, SnpEff: SNPs in the genome of Drosophila melanogaster strain w1118; iso-2; iso-3. Fly.

[85] Milne I, Stephen G, Bayer M, Cock PJA, Pritchard L, Cardle L (2013). Using Tablet for visual exploration of second-generation sequencing data. Brief Bioinform.

[86] Bousfiha A, Jeddane L, Picard C, Ailal F, Bobby Gaspar H, Al-Herz W (2018). The 2017 IUIS Phenotypic Classification for Primary Immunodeficiencies. J Clin Immunol.

[87] Griffith LM, Cowan MJ, Notarangelo LD, Kohn DB, Puck JM, Shearer WT (2016). Primary Immune Deficiency Treatment Consortium (PIDTC) update. J Allergy Clin Immunol.

[88] Rae W, Ward D, Mattocks C, Pengelly RJ, Eren E, Patel SV (2018). Clinical efficacy of a next-generation sequencing gene panel for primary immunodeficiency diagnostics. Clin Genet.

[89] Sun J, Yang L, Lu Y, Wang H, Peng X, Dong X, et al. Screening for primary immunodeficiency diseases by next-generation sequencing in early life. Clin Transl Immunology. 2020;9:e1138. 10.1002/cti2.1138. 10.1002/cti2.1138 PMC7231820 32431812

[90] Rudilla F, Franco-Jarava C, Martínez-Gallo M, Garcia-Prat M, Martín-Nalda A, Rivière J (2019). Expanding the clinical and genetic spectra of primary immunodeficiency-related disorders with clinical exome sequencing: expected and unexpected findings. Front Immunol.

[91] Ng PC, Henikoff S (2003). SIFT: Predicting amino acid changes that affect protein function. Nucleic Acids Res.

[92] Adzhubei I, Jordan DM, Sunyaev SR. Predicting functional effect of human missense mutations using PolyPhen-2. Curr Protoc Hum Genet 2013;Chapter 7:Unit7.20. 10.1002/0471142905.hg0720s76. 10.1002/0471142905.hg0720s76 PMC4480630 23315928

[93] Kircher M, Witten DM, Jain P, O’Roak BJ, Cooper GM, Shendure J (2014). A general framework for estimating the relative pathogenicity of human genetic variants. Nat Genet.

[94] Fadista J, Oskolkov N, Hansson O, Groop L (2017). LoFtool: a gene intolerance score based on loss-of-function variants in 60 706 individuals. Bioinformatics.

[95] McLaren W, Gil L, Hunt SE, Riat HS, Ritchie GRS, Thormann A (2016). The ensembl variant effect predictor. Genome Biol.

[96] Richards S, Aziz N, Bale S, Bick D, Das S, Gastier-Foster J (2015). Standards and guidelines for the interpretation of sequence variants: a joint consensus recommendation of the American College of Medical Genetics and Genomics and the Association for Molecular Pathology. Genet Med.

[97] Kopanos C, Tsiolkas V, Kouris A, Chapple CE, Albarca Aguilera M, Meyer R (2019). VarSome: the human genomic variant search engine. Bioinformatics.

[98] 1000 Genomes Project Consortium, Auton A, Brooks LD, Durbin RM, Garrison EP, Kang HM, et al. A global reference for human genetic variation. Nature. 2015;526:68–74. 10.1038/nature15393. 10.1038/nature15393 PMC4750478 26432245

[99] Lek M, Karczewski KJ, Minikel EV, Samocha KE, Banks E, Fennell T (2016). Analysis of protein-coding genetic variation in 60,706 humans. Nature.

[100] Karczewski KJ, Francioli LC, Tiao G, Cummings BB, Alföldi J, Wang Q (2020). The mutational constraint spectrum quantified from variation in 141,456 humans. Nature.

[101] Chen S, Francioli LC, Goodrich JK, Collins RL, Kanai M, Wang Q, et al. A genome-wide mutational constraint map quantified from variation in 76,156 human genomes. bioRxiv. 2022:2022.03.20.485034. 10.1101/2022.03.20.485034.

[102] Karczewski KJ, Weisburd B, Thomas B, Solomonson M, Ruderfer DM, Kavanagh D (2017). The ExAC browser: displaying reference data information from over 60 000 exomes. Nucleic Acids Res.

[103] Jian X, Boerwinkle E, Liu X (2014). In silico tools for splicing defect prediction: a survey from the viewpoint of end users. Genet Med.

[104] Richmond T (2000). Prediction of intron splice sites. Genome Biol.

[105] Wang M, Marín A (2006). Characterization and prediction of alternative splice sites. Gene.

[106] Yeo G, Burge CB (2004). Maximum entropy modeling of short sequence motifs with applications to RNA splicing signals. J Comput Biol.

[107] Reese MG, Eeckman FH, Kulp D, Haussler D (1997). Improved splice site detection in Genie. J Comput Biol.

[108] Cartegni L, Wang J, Zhu Z, Zhang MQ, Krainer AR (2003). ESEfinder: A web resource to identify exonic splicing enhancers. Nucleic Acids Res.

[109] Edge P, Bafna V, Bansal V (2017). HapCUT2: robust and accurate haplotype assembly for diverse sequencing technologies. Genome Res.

