# Supplementary material for: Genetic screening in a Brazilian cohort with inborn errors of immunity
Source: BMC Genom Data. 2023 Aug 17;24:47. doi: 10.1186/s12863-023-01148-z (PMC10433585; doi:10.1186/s12863-023-01148-z)
Supplement: Supplementary file 1 — Additional file 1: Figure S1. [file 12863_2023_1148_MOESM1_ESM.pptx]

## Slide 1
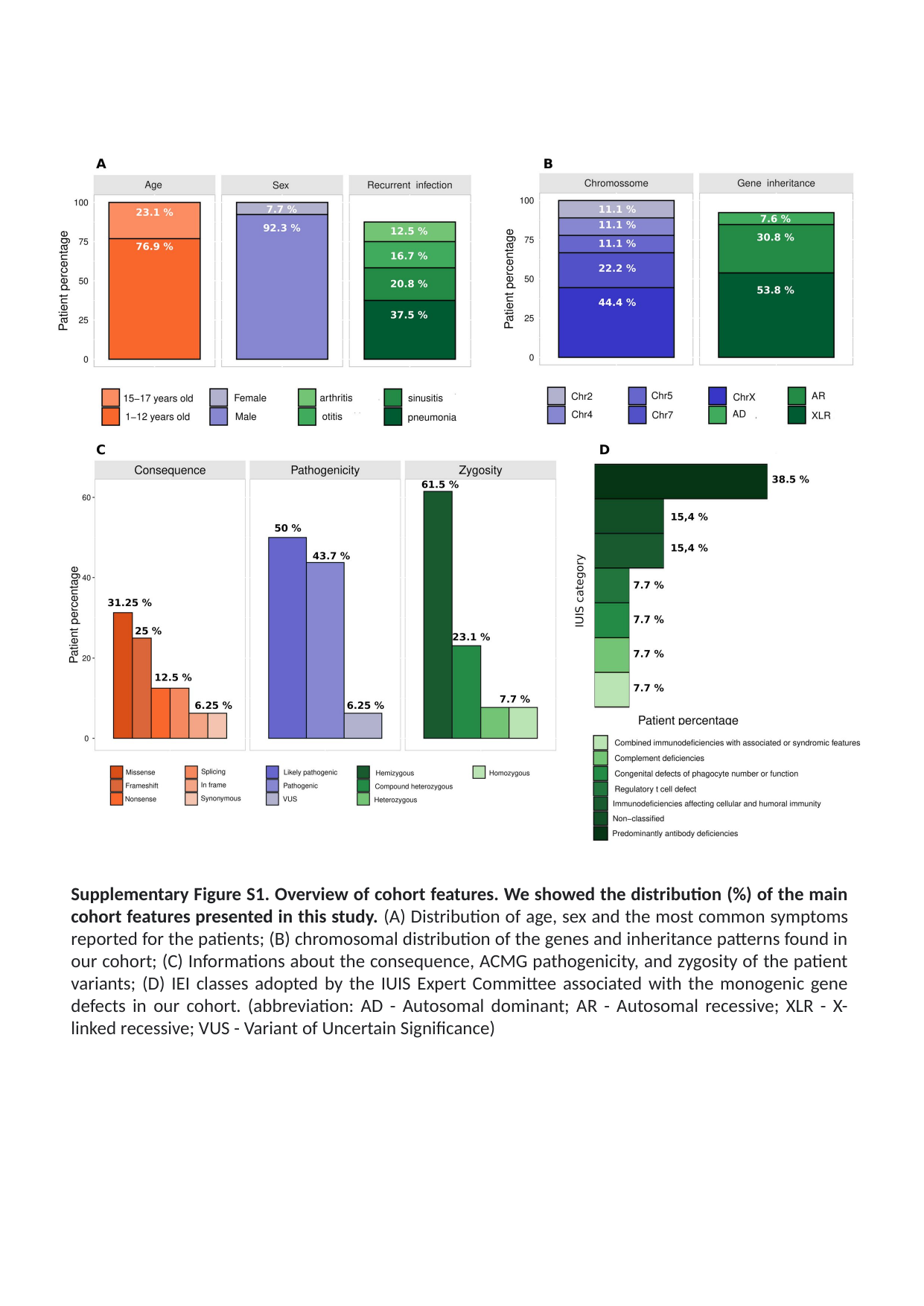

Supplementary Figure S1. Overview of cohort features. We showed the distribution (%) of the main cohort features presented in this study. (A) Distribution of age, sex and the most common symptoms reported for the patients; (B) chromosomal distribution of the genes and inheritance patterns found in our cohort; (C) Informations about the consequence, ACMG pathogenicity, and zygosity of the patient variants; (D) IEI classes adopted by the IUIS Expert Committee associated with the monogenic gene defects in our cohort. (abbreviation: AD - Autosomal dominant; AR - Autosomal recessive; XLR - X-linked recessive; VUS - Variant of Uncertain Significance)
