# Supplementary material for: Genetic screening in a Brazilian cohort with inborn errors of immunity
Source: BMC Genom Data. 2023 Aug 17;24:47. doi: 10.1186/s12863-023-01148-z (PMC10433585; doi:10.1186/s12863-023-01148-z)
Supplement: Supplementary file 2 — Additional file 2: Figure S2. [file 12863_2023_1148_MOESM2_ESM.pptx]

## Slide 1
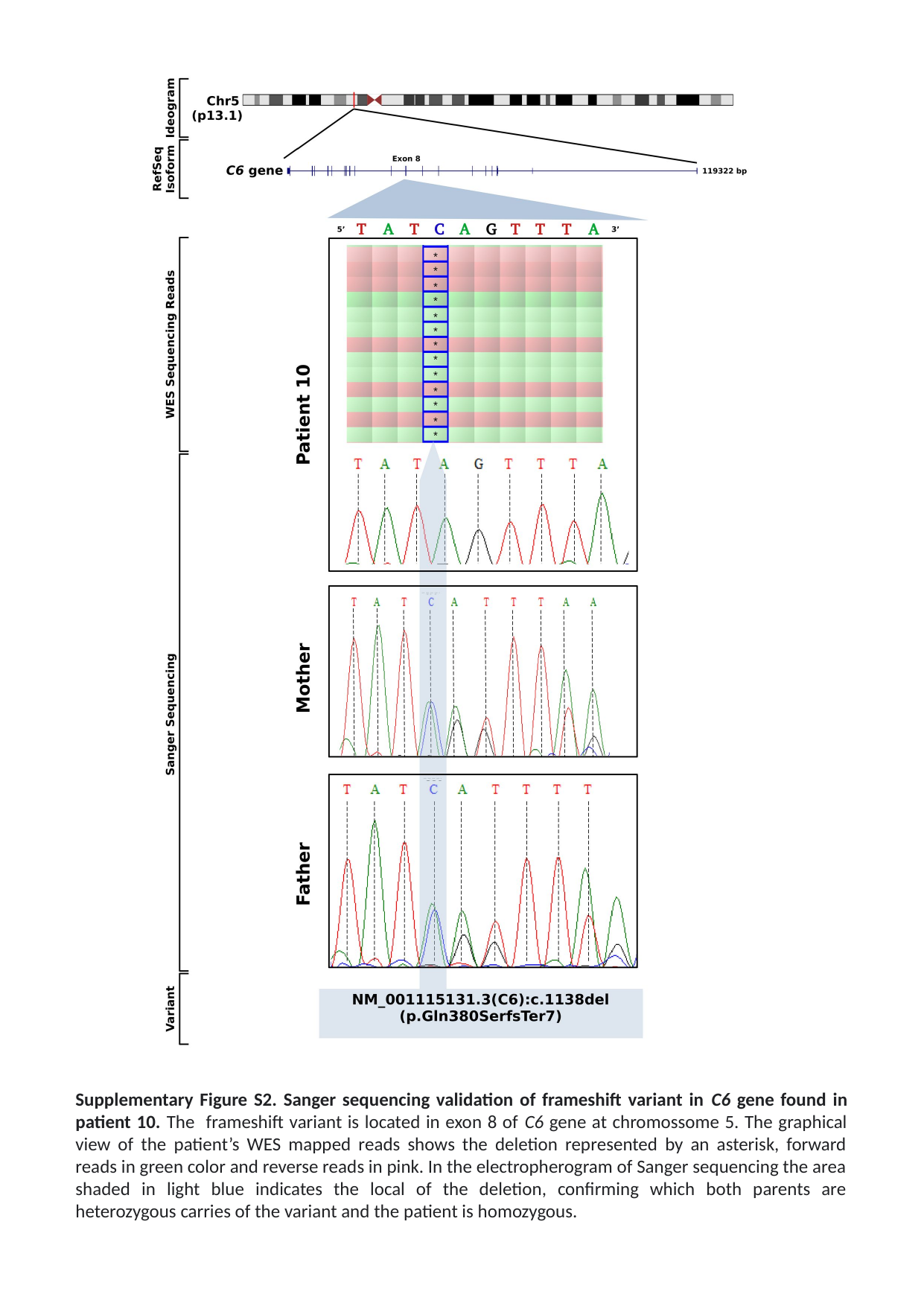

Supplementary Figure S2. Sanger sequencing validation of frameshift variant in C6 gene found in patient 10. The frameshift variant is located in exon 8 of C6 gene at chromossome 5. The graphical view of the patient’s WES mapped reads shows the deletion represented by an asterisk, forward reads in green color and reverse reads in pink. In the electropherogram of Sanger sequencing the area shaded in light blue indicates the local of the deletion, confirming which both parents are heterozygous carries of the variant and the patient is homozygous.
