# Supplementary material for: Genetic screening in a Brazilian cohort with inborn errors of immunity
Source: BMC Genom Data. 2023 Aug 17;24:47. doi: 10.1186/s12863-023-01148-z (PMC10433585; doi:10.1186/s12863-023-01148-z)
Supplement: Supplementary file 3 — Additional file 3: Figure S3. [file 12863_2023_1148_MOESM3_ESM.pptx]

## Slide 1
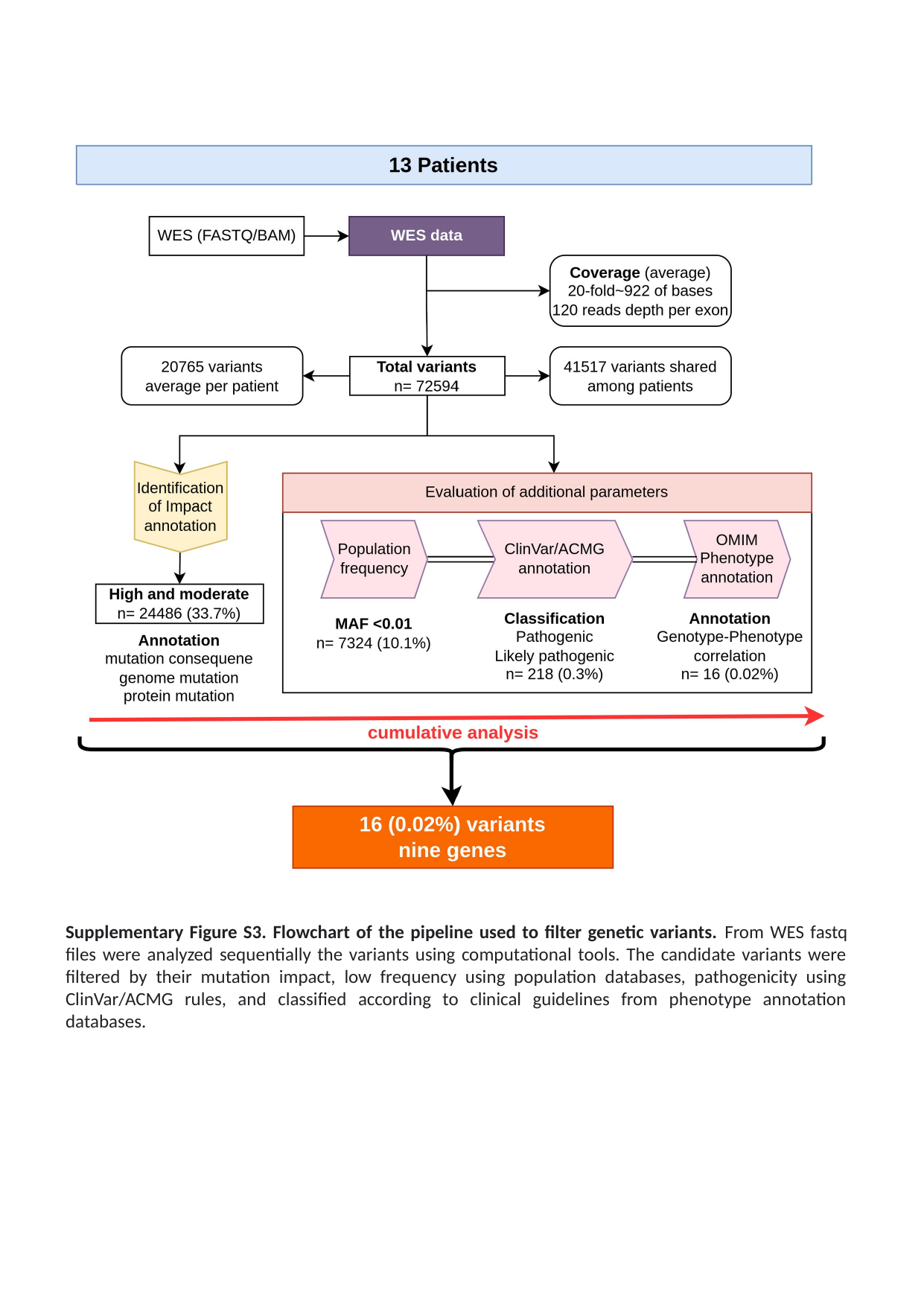

Supplementary Figure S3. Flowchart of the pipeline used to filter genetic variants. From WES fastq files were analyzed sequentially the variants using computational tools. The candidate variants were filtered by their mutation impact, low frequency using population databases, pathogenicity using ClinVar/ACMG rules, and classified according to clinical guidelines from phenotype annotation databases.
